# Vector competence of human-biting ticks *Ixodes scapularis*, *Amblyomma americanum* and *Dermacentor variabilis* for Powassan virus

**DOI:** 10.1186/s13071-021-04974-1

**Published:** 2021-09-09

**Authors:** Rohit Sharma, Duncan W. Cozens, Philip M. Armstrong, Douglas E. Brackney

**Affiliations:** 1 Center for Vector Biology & Zoonotic Diseases, The Connecticut Agricultural Experiment Station, 123 Huntington St, New Haven, CT 06511 USA; 2 Deptartment of Environmental Sciences, The Connecticut Agricultural Experiment Station, 123 Huntington St, New Haven, CT 06511 USA

**Keywords:** Powassan virus, Vector competence, *Ixodes scapularis*, *Dermacentor variabilis*, *Amblyomma americanum*

## Abstract

**Background:**

Powassan virus (POWV; genus *Flavivirus*) is the sole North American member of the tick-borne encephalitis sero-complex and an increasing public health threat in the USA. Maintained in nature by *Ixodes* spp. ticks, POWV has also been isolated from species of other hard tick genera, yet it is unclear if these species can serve as vectors. *Dermacentor variabilis* and *Amblyomma americanum* share geographic and ecologic overlap with *Ixodes* spp. ticks and POWV transmission foci, raising the possibility that POWV could become established in these tick species and leading to range expansion and increased human risk. Therefore, we assessed the competency of *Ixodes scapularis*, *D. variabilis* and *A. americanum* for POWV lineage II (POWV II).

**Methods:**

Larvae from all three species were co-infested on POWV-infected Balb/c mice. The engorged larvae were allowed to molt to nymphs and screened for the presence of POWV II RNA by reverse transcription-qPCR. Eight infected nymphs from each species were allowed to individually feed on a naïve mouse. Mice were screened for the presence of POWV II RNA to determine infection status.

**Results:**

The results demonstrated that larvae from all three tick species were able to efficiently acquire POWV II via feeding on viremic mice, maintain infection through molting and successively transmit POWV to naïve mice at the nymphal stage at comparable rates across all three species.

**Conclusions:**

Our findings reveal that non-*Ixodes* tick species can serve as competent vectors for POWV and highlight the potential role of these species in the ecology and epidemiology of POWV. Future studies examining the possible implications of these findings on POWV epidemiology and the adaptability of POWV in these new vectors are warranted.

**Graphical abstract:**

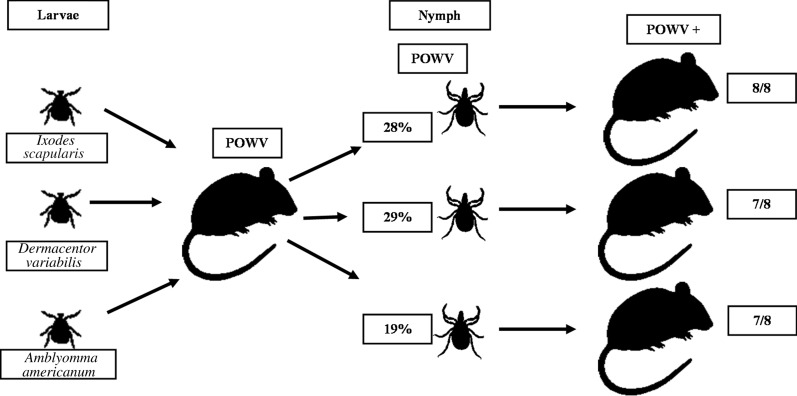

## Background

Powassan virus (POWV; *Flaviviridae*, *Flavivirus*), originally isolated from a fatal case of encephalitis in Ontario, Canada in 1958, is the sole North American member of the tick-borne encephalitis virus (TBEV) sero-complex and is an increasing public health threat in the USA [1–3]. POWV seropositivity has been increasing in deer populations for decades, and the incidence rate of neuroinvasive human POWV cases has nearly tripled since 1998 [4, 5]. Human infections occur through the bite of an infected tick, typically resulting in flu-like symptoms; however, some individuals will develop encephalitis, often resulting in life-long neurologic disability or death. Even more troubling is the rate of transmission; unlike other tick-borne pathogens (e.g. *Borrelia burgdorferi*), which are transmitted over extended periods of tick feeding, POWV can be transmitted to individuals almost instantaneously after tick attachment [6]. Despite the increasing threat, there are no vaccines or therapeutics, and very little is known about POWV evolution, epidemiology, transmission and pathogenesis. Answering these basic questions will be critical to countering this threat and eventually reducing POWV disease risk.

POWV consists of two genetic lineages (POWV I and POWV II [deer tick virus]) that are maintained in distinct transmission cycles [7, 8]. POWV I is maintained in a transmission cycle between *Ixodes marxi* and *Ixodes cookei* ticks and small to medium-size rodents [9, 10]. These tick vectors are generally highly host specific and rarely encountered by humans. Conversely, POWV II is maintained in a transmission cycle between the commonly human-biting *Ixodes scapularis* (black-legged or deer tick) and *Peromyscus leucopus* (white-footed mouse) [11, 12]. Results of molecular epidemiological studies suggest that the two lineages diverged as a result of their distinct transmission cycles [13], which occurs when an arbovirus encounters and adapts to a new arthropod vector and eventually becomes fixed within the novel transmission cycle. Evidence suggests that TBEV sero-complex members occasionally experience periods of rapid diversification during adaptation to a new tick species, possibly through hybrid species, thereby explaining the radiation of TBEV subtypes throughout Europe and Asia [14]. The recent identification of a hybrid of *I. scapularis* and *I. cookei* suggest that POWV I and II may have experienced analogous evolutionary paths [15].

There is evidence that non-*Ixodes* species could also be involved in POWV transmission as the virus has been isolated from field-collected *Dermacentor* and *Haemaphysalis* species ticks [16, 17]. This is not surprising considering that many other tick-borne viruses have been isolated from a number of species from hard-tick genera not considered to be their primary vectors [18]. Other known human-biting ticks, such as *Dermacentor variabilis* and *Amblyomma americanum*, are widely distributed in the eastern USA and share varying degrees of geographic and ecologic overlap with *Ixodes* spp. ticks and POWV transmission foci. For example, *D. variabilis* and *I. scapularis* can be found co-infesting the nests of *Peromyscus* mice in POWV endemic regions and *I. scapularis* and *A. americanum* both readily feed on deer [19]. Such co-occurrences could facilitate cross-species transmission via co-feeding. Previous studies have found that uninfected ticks feeding in close proximity to TBEV infected ticks could become infected themselves [20]. Consequently, there exists the possibility that POWV could become established in other non-*Ixodes* species; however, it is unclear if *D. variabilis* and *A. americanum* can become infected and transmit POWV.

To address these unknowns, we compared the vector competency of *I. scapularis*, *A. americanum* and *D. variabilis* for POWV II*.* We demonstrate that all three tick species can efficiently acquire POWV II while feeding on viremic Balb/c mice at the larval stage, maintain infection through molting and transmit POWV II to the naïve mice via blood-feeding at the nymphal stage. These findings highlight the potential of POWV to emerge in non-*Ixodes* hard tick species.

## Methods

### Ticks and animals

Larvae from all three tick species were obtained from the Oklahoma State University Tick Rearing Facility (Stillwater, OK, USA). *Ixodes scapularis* and *D. variabilis* colonies have been maintained at the facility since 1991 and 1972, respectively, and originated from a natural population of engorged females collected from Payne County, Oklahoma. The *A. americanum* colony has been maintained since 1976 and started with engorged females collected from Cherokee County, Oklahoma. Tick larvae were placed in glass rearing vials covered with nylon mesh and perforated plastic caps. The vials were kept within a sealed bell jar containing water at room temperature, at 98% relative humidity and under a 16/8-h light/dark cycle. Six-week-old female Balb/c mice were supplied by the Charles River Laboratory (Wilmington, MA, USA) and maintained in a BSL3 laboratory as per the Institutional Animal Care and Use Committee (Connecticut Agriculture Experiment Station/Institutional Animal Care and Use CommitteeI (ACUC)/Office of Laboratory Animal Welfare; AWAN-A4050-01). All handling and manipulation involving ticks and animals were performed according to the approved protocols.

### Cell culture, virus maintenance and titrations

Baby hamster kidney (BHK-21) cells were grown in Dulbecco’s modified Eagle’s medium (DMEM) supplemented with 10% fetal calf serum (Gibco, Dublin, Ireland) and 1% penicillin–streptomycin solution (Gibco; Dublin, Ireland) at 37° with 5% CO_2_. POWV lineage II (strain P0183), derived from a field-collected adult *I. scapularis* from Connecticut during 2010, was amplified on BHK-21 clone 15 cells . Virus titers were determined by plaque assay on BHK-21 clone 15 cells using the methyl cellulose approach. Briefly, 95% confluent BHK-21 cells in 12-well plates were infected with diluted virus stock for 1 h at room temperature, following which the virus inoculum was removed, and a 1% methyl cellulose solution diluted in 10% complete MEM media was added to each well. The plates were then incubated at 37 °C with 5% CO_2_ for 5 days prior to fixation with 7.5% formaldehyde solution. The cells were then stained with 1% Crystal violet dye solution and plaque-forming units (PFUs) were enumerated.

### Tick infection and virus transmission studies

Mice were intraperitoneally injected with 2.6 × 10^2^ PFUs of POWV diluted in endotoxin-free phosphate-buffered saline (PBS). Immediately after inoculation individual mice (*n* = 8) were immobilized within a wire mesh restrainer, and  approximately 50 tick larvae per species (total of approx. 150 tick larvae per mouse) were placed on the nape of each mouse using a fine brush. Each mouse was infested with all three tick species concomitantly to eliminate effects on infection rates that may arise from variable mouse viremias. Restrainers were then wrapped with a piece of paper and kept undisturbed for 1 h. Each mouse was carefully transferred to a standard aluminum cylindrical cage that was suspended over a plastic pan filled with water. Engorged larvae were collected from the water pans upon detachment 3–6 days post-infestation (dpi) and placed in rearing vials to allow for molting. Mice were euthanized after completion of the feeding experiment.

At approximately 3 weeks post-molting, emerged nymphs were cold-anesthetized, identified to species and the third right leg of each nymph removed to test POWV-II infection status by reverse transcription quantitative PCR (RT-qPCR). To assess the horizontal transmission potential of each species, infected nymphs obtained from the larval feeding experiments were individually placed on the naïve mice (*n* = 8 mice/tick species) using the feeding capsule method as previously described. Briefly, mice were anesthetized with ketamine (0.15 mg/gm) and their napes shaved. A 2-ml screwed cap tube cut in half was secured to each mouse using a melted mixture of gum rosin with bee wax (4:1). Individual ticks were placed inside the tubes and the screw caps fastened. To allow for air exchange, a small puncture covered by cotton was introduced into the cap. Upon engorgement, detached nymphs were collected and maintained until adults emerged. Mice were euthanized by overexposure to CO_2_ within 10 days of tick attachment or at the first signs of severe disease, as indicated by tremors and hind limb paralysis. Mice carcasses were frozen for future processing and at the same time point brain tissue and spleen were retrieved and RNA extracted as described below.

### RNA isolation and RT-qPCR

Total RNA was isolated by using the Mag-Bind Viral RNA 96 kit (Omega Biotek; Norcross, GA, USA) on Kingfisher flex (Thermo Fisher Scientific; Waltham, MA, USA). Briefly, tick leg or mice brain or spleen tissues were placed in tubes containing 250 µl PBS-G (PBS with 0.5% gelatin, 30% rabbit serum and 1% 100× antibiotic–antimycotic [10,000 μg/ml of streptomycin and 25 μg/ml of amphotericin B]) and a stainless-steel ball bearing and macerated using a mixer mill. A 50-µl aliquot of homogenate was used for the RNA extraction following the protocol of the kit’s manufacturer (Omega Biotek). RT-qPCR was performed on a Bio-Rad C1000 Touch system with a CFX96 optical module using the Reliance One-Step Multiplex RT-qPCR Supermix (Bio-Rad Laboratories, Inc.; Hercules, CA, USA) with the following cycline parameters: reverse transcription at 55 °C, 15 min; then 95 °C, 10 min; followed by 95 °C/15 s and 60 °C/30 s for 40 cycles. The primer/probe set used to target the 3′-untranslated regions of POWV II was as previously reported [21]. Quantification of POWV II genome equivalents was performed using a previously developed assay with the same reagents and cycling parameters as described above [22].

### Data analysis

The species composition of molted nymphs was calculated as the percentage of molted nymphs from each species from the total number of molted nymphs. The rate of infection was calculated based on the percentage of nymphs testing positive for the virus within the total number of nymphs tested from each species. The efficiency of horizontal transmission of POWV II was represented as the number of mice that acquired infection and became ill, as confirmed by post-mortem detection of virus in mouse brain and/or spleen tissue by the RT-qPCR. Fisher’s exact test was used for all pairwaise comparisons. Analyses were conducted using the Prism software package version 8.0 (GraphPad Software Inc., San Diego, CA, USA), and a *P* ≤ 0.05 was considered to be significant.

## Results

### Molting success, species composition of emerged nymphs and POWV-II transmission

**Fig. 1:**
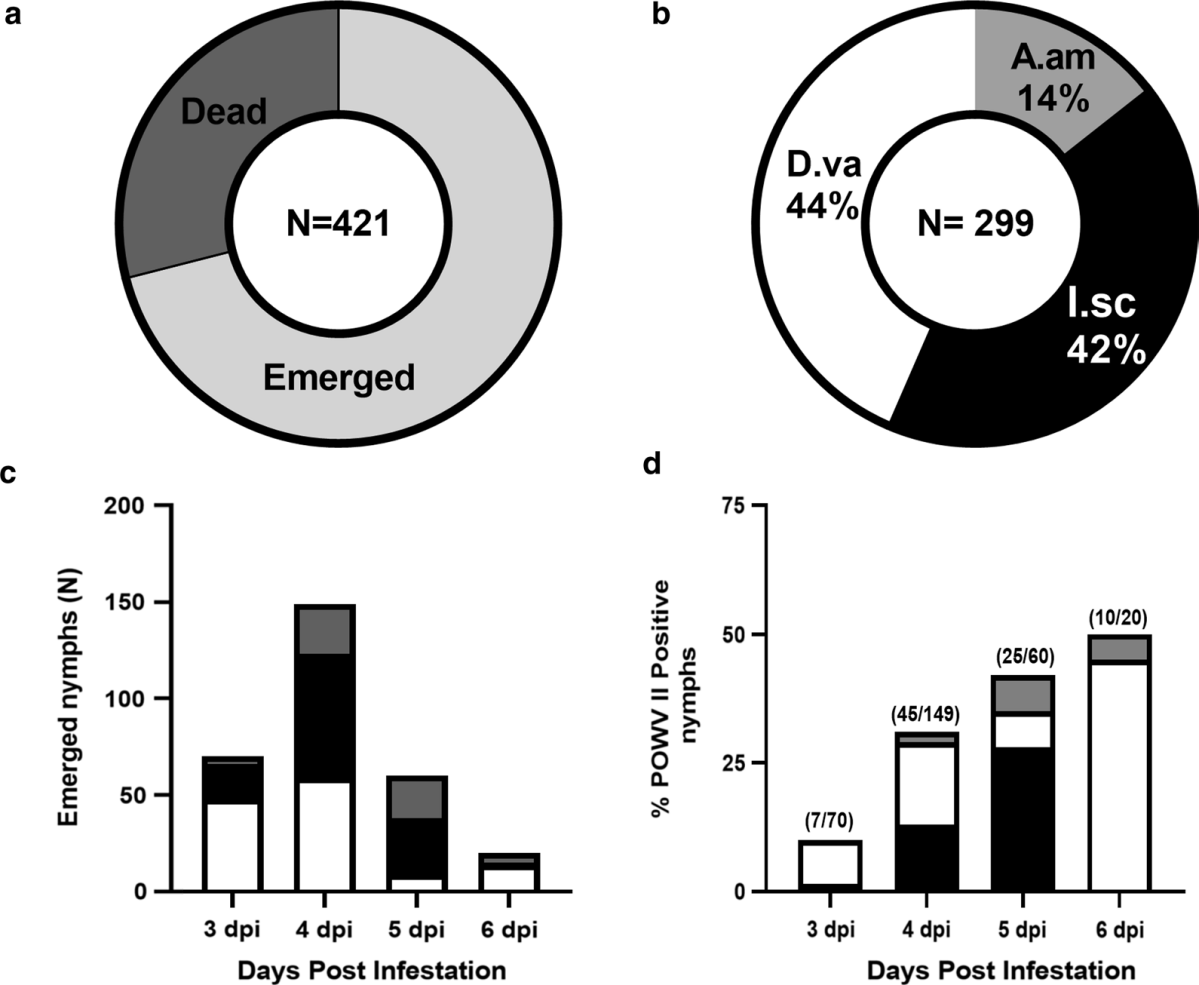
Feeding success of *Ixodes scapularis*, *Dermacentor variabilis* and *Amblyomma americanum* ticks on Powassan virus lineage II (POWV II)-infected Balb/c mice. **a** Combined molting success of all engorged larvae is represented as the percentage of emerged nymphs divided by the total number of engorged larvae (*n* = 421). **b** Species composition of emerged nymphs represented as the percentage of each species divided by the total number of emerged nymphs.* I.sc*  *I. scapularis*,* D.va*  *D. variabilis*,* A.am*  *A. americanum*. **c** Species composition at detachment (number of days post-infestation). Bars: black represents  *I. scapularis*; white,  *D. variabilis*; gray,  *A. americanum*. **d** Percentage of POWV II-positive emerged nymphal ticks by number of days post-infestation at detachment broken down by tick species (coloration in bar as in** c**)

**Fig. 2:**
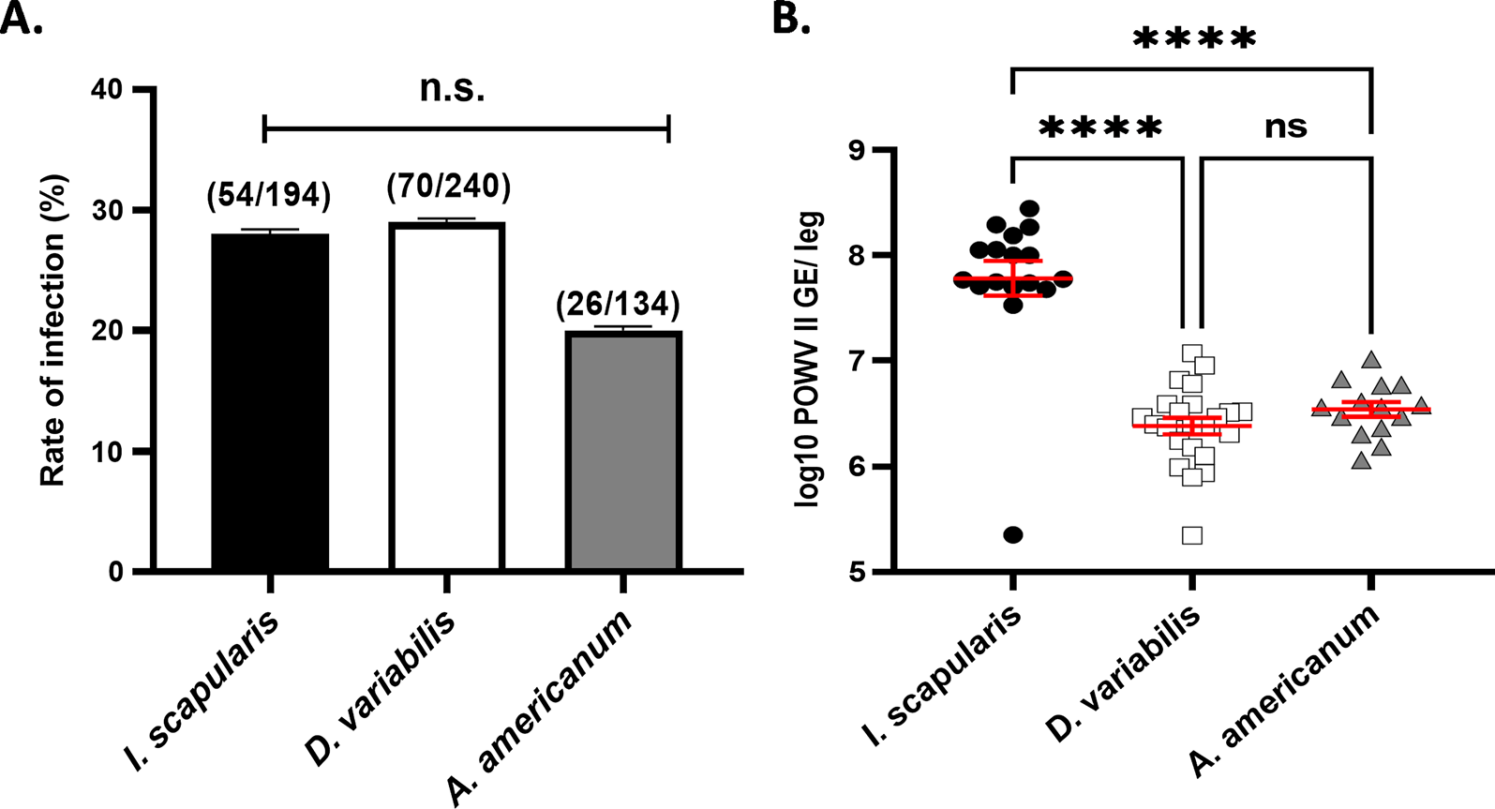
POWV II infection rates of nymphal *I. scapularis*, *D. variabilis* and *A. americanum* ticks. **a** Infection rates determined by dividing the number of infected individuals by the total number tested, by species. Data were statistically examined using a Fisher’s Exact Test. **b** POWV II genome equivalents (*GE*) per infected nymphal leg. Statistical significance was tested using a one-way ANOVA and Tukey’s multiple comparisons test (****P < 0.0001)

To examine the competency of *I. scapularis*, *D. variabilis* and *A. americanum* to serve as vectors for POWV II, Balb/c mice were intraperitoneally injected with POWV II and co-infested with each of the three tick species. Upon detachment and molting, POWV II-positive nymphs were identified by screening the leg from each tick by RT-qPCR. During the initial infection, all mice showed clinical signs of progressive disease, and most engorged larvae detached between 3 and 6 dpi. In total, we recovered 421 engorged larvae, which were separated and categorized based on the day of collection post-infestation. Because it can be difficult to accurately differentiate species of engorged larvae, species identification occurred after molting. Among the engorged larvae, 299 (71%) successfully molted into nymphs (Fig. 1a). Of these, 130 were *D. variabilis* (44%), 126 were *I. scapularis* (42%) and 43 were *A. americanum* (14%) (Fig. 1b). These findings are not particularly surprising given that small rodents are the preferred natural host of *D. variabilis* and *I. scapularis* larvae, but not of *A. americanum* larvae. Examination of attachment time by species revealed no significant difference in feeding/attachment duration (Fig. 2c). Emerged nymphs from all three tick species represented fed larvae recovered each day between 3 and 6 dpi (Fig. 1c), with the highest numbers recovered on 4–5 dpi. The proportions of emerged nymphs infected with POWV II were highest for larvae recovered on 6 dpi (Fig. 1d). Ticks collected and testing positive for POWV II at 6 dpi were almost exclusively *D. variabilis*. Taken together, these results confirm that larvae from all three tick species feed upon mice in a laboratory setting with varying degrees of success and typically require 3–6 days to fully engorge.

### POWV II infection rates of emerged nymphs

To ascertain the infection rates of each of the three tick species, we utilized data from the total number of nymphs (*n* = 568) emerged from multiple larval feeding experiments, carefully removed the third right leg of all emerged nymphs, extracted RNA and then assayed the RNA for the presence of POWV II RNA by RT-qPCR. Nymphal infection rates were highest for *D. variabilis* (29.2%, 70/240) and *I. scapularis* (27.8%, 54/194), the latter being the primary vector of POWV II (Fig. 2a). *Amblyomma americanum* had the lowest rate of nymphal infection at 19.4% (26/134). However, differences in the rate of infection between the three tick species were not statistically significant (*I. scapularis* vs *D. variabilis*, *P* = 0.83; *I. scapularis* vs *A. americanum*, *P* = 0.2; *D. variabilis* vs *A. americanum*, *P* = 0.11) (Fig. 2a). Viral burden was significantly higher in *I. scapularis* compared to both *D. variabilis* and *A. americanum* (*I. scapularis* vs *D. variabilis*, *P* < 0.0001; *I. scapularis* vs *A. americanum*, *P* < 0.0001) (Fig. 2b). Despite differing viral burdens, these results demonstrate that all three species are able to acquire infection from a viremic mouse and maintain the infection transstadially through molting.

### Horizontal transmission of POWV II to the naïve mice

Infected nymphs were tested for their potential to transmit POWV II to naïve mice via horizontal transmission. Eight infected nymphs from each of the three species were individually placed on naïve mice and allowed to feed for 3–5 days. Mice were monitored during this time and euthanized either on day 10 or at the first signs of illness. Signs of infection emerged between 4 and 10 dpi and included loss of appetite, lethargy, disorientation and hind leg paralysis. Because some mice recovered from minor signs of infection (i.e. lethargy and loss of appetite), the brains and spleen from all mice were analyzed by RT-qPCR for the presence of POWV II RNA. The results confirmed the presence of POWV II in brain and/or spleen tissues of individual nymph-infested mice (*I. scapularis*, 8/8; *D. variabilis*, 7/8; *A. americanum*, 7/8) (Table 1).

**Table 1:** Horizontal transmission of Powassan virus lineage II to mice by *Ixodes scapularis*, *Dermacentor variabilis* and *Amblyomma americanum*

Tick species	Exposed mice (*n*)	Mice with signs of infection (*n*)	POWV II Infection (*n*)^a^
*Ixodesscapularis*	8^b^	8	8
*Dermacentorvariabilis*	8^b^	8	7
*Amblyommaamericanum*	8^b^	8	7

*POWV II* Powassan virus lineage II

^a^Data obtained from RT-qPCR analysis of brain and/or spleen tissues of exposed mice

^b^6 (fully engorged) + 2 (partially fed) nymphs recovered from feeding capsules

## Discussion

In this study, we evaluated the ability of *I. scapularis*, *D. variabilis* and *A. americanum* ticks to become infected and transmit POWV II. We found that all three tick species were equally capable of acquiring POWV II as larvae and transmitting virus as nymphs. These findings are consistent with field observations that tick-borne viruses (tiboviruses) can often infect numerous genera of hard ticks, including non-primary vector species in the field. For example, the primary vectors for TBEV are *Ixodes* spp. ticks but the virus has been detected in field-collected *Dermacentor reticulatus* [23]. Similarly, a virus closely related to POWV, which is primarily associated with *Ixodes marxi* and *I. cookei*, has been isolated from *Dermacentor andersoni* [17]. Interestingly, we observed that POWV II RNA levels were significantly higher in nymphs of the primary vector *I. scapularis*. The biological significance is unclear, but similar findings have been observed for field-collected primary and secondary vectors of TBEV [24]. Nevertheless, these findings suggest that tiboviruses can and will readily infect other hard tick species given the opportunity; however, the detection of tiboviruses from any tick species does not necessarily imply that it is a vector. To demonstrate competency, it must first be established that the tick species can acquire the virus via feeding on a viremic host, transstadially pass the virus from one life stage to the next and transmit the virus in a subsequent feeding event [25]. Despite their public health importance, vector competence studies on tiboviruses have been limited. The results from those that have been performed tend to support the observations from the field. For example, Chernesky et al. demonstrated that *D. andersoni* was a vector for POWV I [26]. Similarly, species from several hard tick genera, including *Ixodes* spp., *Dermacentor* spp. and *Haemaphysalis* spp., were proven to serve as vectors for TBEV, and *Haemaphysalis* spp.,* Rhipicephalus* spp. and *Amblyomma* spp. were shown to support infection and transmission of Thogoto virus [20, 25, 27]. This broad vector range observed for many tiboviruses can have significant public health implications. Work with chikungunya virus, a mosquito-borne alphavirus, has demonstrated that replication and transmission in new vector species can impose unique selective pressures that can affect the ecology and transmissibility of the virus [28]. Further establishment of tiboviruses in non-primary vectors could lead to new viral subtypes with varying ecologies and disease phenotypes, as has been proposed for the radiation of TBEV subtypes [14]. Thus, based on our findings and the examples from other vector–virus systems, non-*Ixodes* tick species could serve as potential vectors for POWV II, leading to range expansion and increased public health risk.

The POWV transmission cycle is thought to be perpetuated through three main modes of transmission: direct feeding on a viremic host; vertically from mother to offspring; and co-feeding (transmission from an infected tick to a naïve tick by feeding in close proximity to one another). Direct feeding is a common mode for initiating infection in the laboratory, as was done in this study. Balb/c mice are highly susceptible and produce a viremia sufficient to transmit the virus to attached ticks; however, *Peromyscus leucopus*, the purported reservoir host, does not support levels of replication conducive for transmission [29]. While it is possible that other unidentified reservoir hosts exist, co-feeding and vertical transmission are the most likely modes of maintenance in nature [30]. Consequently, ecologic overlap would be a prerequisite for POWV II emergence in *D. variabilis* and *A. americanum*. All three species are geographically distributed in much of the eastern half of the USA, reside in similar habitats and can often be collected from the same survey site [31, 32]. In addition, all three species share many of the same vertebrate hosts. *Dermacentor variabilis* and *I. scapularis* immatures can often be found parasitizing the same rodent hosts whereas all stages of *A. americanum* and *I. scapularis* feed readily on deer [19, 32]. This overlap increases the likelihood of POWV II entering *D. variabilis* or *A. americanum* populations via co-feeding or viremic transmission.

In this study we assessed the infection rates of POWV II in three species of hard ticks by allowing larvae to feed on an infectious mouse, collecting the engorged larvae and screening the emergent nymphs for POWV II infection. This approach does not discern the efficiency of transstadial transmission by comparing the proportion of larvae and resulting nymphs that acquired and maintained POWV infection through the molt. However, testing engorged larvae prior to molting is also problematic because remnants of the infectious blood meal remain in the tick for weeks and, therefore, differentiating virus in the blood from an actual infection can be difficult. Although neither approach for assessing infection rates is ideal, there are data to suggest that transstadial transmission of POWV is 100% efficient in *I. scapularis* [22, 33]. In fact, all POWV II nymphal ticks retained infection as adults in this experiment (data not shown). Thus, screening nymphs to estimate infection rates should be highly accurate. Another factor impacting infection rates is the duration of tick feeding. We found that ticks that remained attached longer to the infected mouse were more likely to become infected (Fig. 1d). In this study, following inoculation of POWV II, mice were immediately infested with ticks. Engorged ticks were recovered between 3 and 6 dpi, with the highest proportion of recovery on 4–5 dpi. Infection rates for all three tick species was 19–29%; however, the rates of infection differed between days post-infestation, with the proportion of infected ticks being highest at 6 dpi. Because peak detachment day does not correspond with peak infection day, it is possible that if the mice were infested 1–2 days post-infection the overall infection rates might be significantly higher. This observation highlights some of the potential variables influencing tibovirus vector competence studies and should be considered in future studies.

In conclusion, we demonstrate that all three tick species evaluated acquire infection and transmit POWV to mice at similar rates. These findings have important implications for understanding the public health risk posed by these tick species. Human cases of POWV encephalitis have become increasingly prevalent in eastern USA where *Ixodes scapularis* is the dominant vector. Our findings suggest that other human-biting ticks, specifically *A. americanum* and *D. variabilis*, could also acquire infection and serve as vectors given the extent of ecological overlap with the primary vector. Future investigations are needed to evaluate their potential roles in supporting POWV transmission. This includes studies to: (i) estimate the prevalence of viral infection in these tick species in POWV endemic sites; (ii) evaluate viral populations for adaptive genetic changes when infecting new tick species; and (iii) assess whether these species can maintain POWV infection by co-feeding and vertical transmission.

## Data Availability

All data generated or analyzed during this study are included in this published article.
